# Effects of the Timing of Carbohydrate Intake on Metabolism and Performance in Soccer Players

**DOI:** 10.3390/nu15163610

**Published:** 2023-08-17

**Authors:** Ki-Woong Noh, Jung-Hwan Oh, Sok Park

**Affiliations:** 1 Institute of Sports Medicine & Science, Kwangwoon University, Seoul 01897, Republic of Korea; shrldn123@kw.ac.kr; 2 Department of Kinesiology and Community Health, University of Illinois at Urbana-Champaign, Champaign, IL 61820, USA; 3 Department of Convergence Sports Science, Kwangwoon University, Seoul 01897, Republic of Korea

**Keywords:** carbohydrate, intake timing, soccer-specific, performance, intermittent exercise

## Abstract

This study aims to provide information to improve the performance of athletes comparing the effects of carbohydrate–electrolyte intake before and during exercise on metabolism and performance in soccer players. The study had a single-blind cross-over design. Drust’s protocol is a soccer-specific intermittent exercise test. The carbohydrate–electrolyte intake experiments were divided into three timings: first, pre-exercise; second, half-time; and third, mixed. Eight participants were included in the data analysis (age: 21.32 ± 1.19 years; BMI: 22.69 ± 1.91 kg/m^2^; height: 176.5 ± 7.52 cm; weight: 69.5 ± 9.18 kg; V_max_: 16.75 0.71 km/h). The results of the mixed test showed a significantly lower respiratory exchange ratio than those of the placebo and half-time tests (*p* < 0.05). The mixed test showed significantly more fat oxidation than the half-time test (*p* < 0.05). The running times are placebo (422.13 ± 133.44 s) and mixed (677.38 ± 217.75 s), and the distances are placebo (1577.25 ± 517.02 m) and mixed (2530.00 ± 832.71 m) (*p* < 0.05). The mixed test showed a significantly lower rating of perceived exertion than the placebo test (*p* < 0.05). Carbohydrate oxidation and heart rate showed no significant differences between the experiments (*p* > 0.05). The exercise protocol in this study showed the metabolic response of soccer players to intermittent high-intensity exercise and subsequent endurance exercise. In conclusion, it can be seen that the intake of carbohydrate–electrolytes improves the performance of soccer players, and the effect varies depending on the timing of carbohydrate–electrolyte intake.

## 1. Introduction

Athletes are constantly looking for ways to maximize their potential and improve their performance. Ergogenic aids are defined as substances that are used to improve performance by increasing energy supply [1]. Ergogenic aids supplement athletes’ insufficient meals, meet the needs of depleted nutrients through high-intensity training, and improve exercise performance by improving endurance, shortening recovery time, and preventing fatigue, disease, and infection [2]. Carbohydrate (CHO) intake pre-exercise, during exercise, and post-exercise plays a critical part in an athlete’s performance. Many studies have consistently proven the ergogenic effects of CHO supplements during continuous moderate- to high-intensity exercise [3]. CHO intake before exercise can improve exercise ability compared to exercise in a fasting state. In addition, CHOs are a significant energy source for performing moderate- and high-intensity exercise [4].

Not all CHOs are equal; some are used more rapidly than others, and those are the ones we need for optimal performance [5]. CHO intake pre-, during, and post-exercise plays a critical part in an athlete’s performance. The timing and amount of CHOs are important when developing a fueling plan. Each athlete should follow a tailored plan based on their body weight, the duration and intensity of the bout of exercise, as well as their gut tolerance. The human body cannot use more than 60 g/h of a single CHO [6]. For exercise lasting less than 30 min, there really is no need to ingest carbohydrates during exercise. However, for events or exercises lasting longer than 30 min, the amount and type of carbohydrates ingested during exercise are dependent on the duration and intensity of the exercise [7]. The ingestion of carbohydrates, even in small amounts, can improve performance during long-term exercise (>2 h) [8]. In addition, ingesting a CHO supplement ten minutes before an exercise will result in better exercise performance [9]. Studies have proven that an intake of 30 g–60 g of CHOs every hour is the most effective in sports with a duration of 60–150 min. Despite studies proving the beneficial effects of CHOs consumed before or during exercise, the optimal intake method and types of CHOs are not well known [10].

Soccer is a high-intensity sport that lasts more than 90 min. Many studies recommend taking CHO supplements because muscle glycogen and blood sugar levels drop to the point of affecting performance during the game. A potential strategy to overcome the limited opportunities to consume CHOs during a soccer match is to administer a highly concentrated CHO–electrolyte solution [11]. The consumption of a CHO–electrolyte solution during prolonged exercise is also beneficial for attenuating dehydration and improving performance. To fuel provision and prevent dehydration, 6–12% CHO–electrolyte beverages are recommended to maintain osmotic balance and decrease soccer-specific fatigue [12]. Practical recommendations on the type of CHO intake and the appropriate CHO–electrolyte intake are required to facilitate gastric emptying and compensate for sweat loss [13].

Studies to improve soccer players’ performance are considered important, and a lot of them are being conducted. In addition, soccer has been found to have an effect on the intake of CHOs, as the results of several studies have shown, and the need for proper CHO intake is being emphasized. Currently, CHO intake during exercise has become a common practice among athletes in many other sports with diverse durations and intensities of exercise. However, there are few studies on the proper timing of CHO intake in soccer-specific exercise. Accordingly, this study aims to provide information to improve the performance of athletes by comparing the effects of CHO–electrolyte intake consumed before and during exercise on metabolism and performance in soccer players.

## 2. Materials and Methods

### 2.1. Participants

Eight male soccer players attending K University in Seoul were recruited as subjects for this study. The method of the study was sufficiently explained to all subjects, and the subjects gave written informed consent to the study, which had been approved by the University of Kwangwoon Bioethics Committee. To safely proceed with the soccer-specific exercise test, a physical activity readiness questionnaire was prepared in advance to determine whether the test could proceed. As a result of the questionnaire, all eight participants were not limited to participating in the experiment. Only one out of the eight participants smoked (20 per week), and five out of the eight consumed alcohol, but the amount was not large (no more than five glasses per week). The general characteristics of the subject are as follows: age, mean ± standard deviation (SD) (21.32 ± 1.19); weight, mean ± SD (69.5 ± 9.18); height, mean ± SD (176.5 ± 7.52); BMI, mean ± SD (22.69 ± 1.91); V_max_, mean ± SD (16.75 ± 0.71).

### 2.2. Experimental Design

**Figure 1:**
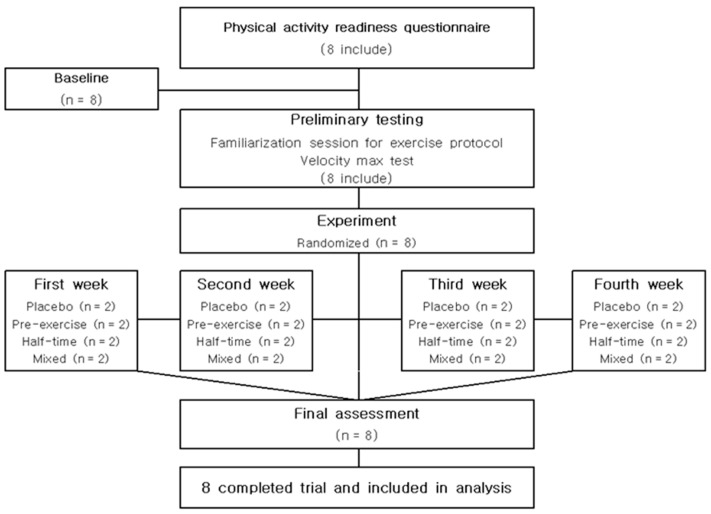
Flow chart in the study.

**Figure 2:**
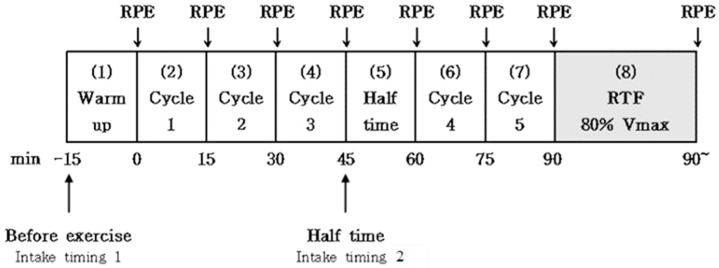
This figure shows a schematic representation of the experimental protocol. RTF: run time fatigue; RPE: ratings of perceived exertion.

All subjects completed four experimental tests. All experimental tests were completed in the same laboratory and under the same environmental conditions. The subjects were instructed to refrain from intense physical activity and were also required to refrain from alcohol, caffeine, and smoking consumption for 24 h before each experimental test [14]. On the day of the experiment, the participants reported to the laboratory after fasting overnight (≥10 h). The study had a single-blind cross-over design. In four experiments (placebo; pre-exercise; half-time; mixed), two subjects participated weekly for each experiment, and eight subjects were randomized (Figure 1). On the day of the experiment, a stable condition was determined for 10 min after athletes arrived at the laboratory, and then they took a CHO–electrolyte solution or a non-CHO–electrolyte placebo within 5 min before the 15 min soccer-specific intermittent exercise test [15]. There was a 15 min rest time 45 min after the start of the exercise, which is similar to the half-time of a soccer match. A five-minute warm-up at 8 km/h followed by five minutes of stretching was performed prior to the test [16]. Respiratory gas and heart rate (COSMED Quark CPET, Rome, Italy) were continuously measured during the soccer-specific exercise tests. Ratings of perceived exertion (RPE) were measured every 15 min on the Borg scale [17], divided into steps 1 to 10 [18] (Figure 2). Respiratory exchange ratio (RER), CHO oxidation, and fat oxidation were expressed by an equation using the measured oxygen consumption (VO_2_) and carbon dioxide production (VCO_2_).

### 2.3. Preliminary Testing

Preliminary testing included a velocity max (V_max_) test and a familiarization session for the high-intensity intermittent exercise protocol. After the preliminary test, each subject went through four experiments at intervals of 7 days or more in a balanced single-blind cross-over design [16]. V_max_ were determined using an incremental running test to exhaustion on a treadmill, accordingly [19]. On the day of the preliminary test, a stable condition was maintained for 10 min after arriving at the laboratory. A five-minute warm-up at 8 km/h followed by five minutes of stretching, was performed prior to the test. The test started at 10 km/h, and the speed was increased by 1 km/h every minute until exhaustion. The measured V_max_ is used to set the speed of the run time fatigue (RTF) cycle in soccer-specific intermittent exercise protocol [20].

### 2.4. Exercise Protocol

**Figure 3:**
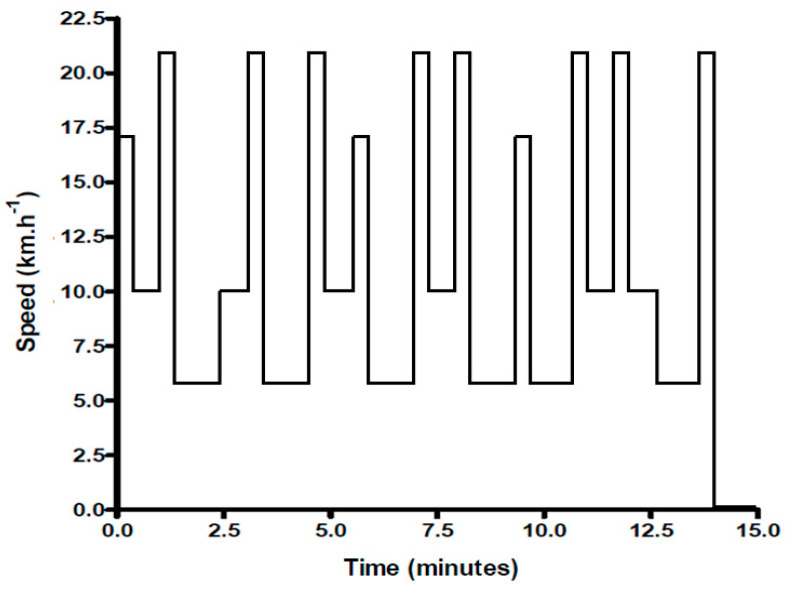
Graphical representation of one 15 min cycle of the soccer−specific intermittent exercise protocol.

The soccer-specific intermittent exercise protocol was applied for the laboratory-based study [11]. This exercise protocol was made to simulate the exercise intensity and physiological demands of soccer match (Drust et al. (2000) [21]). The experimental protocol design was composed of 5 × 15 min identical intermittent exercise cycles, immediately followed by run time to fatigue at 80% V_max_. The total duration of the 5 cycles was 75 min of intermittent exercise interposed with a 15 min rest period at half-time. The subjects were to run to the point of exhaustion, at which time they could not maintain their relative speed (V_max_ 80%) in the last cycle [15]. A treadmill (COSMED T-150, Rome, Italy) and its software (COSMED Quark CPET Omnia, version 1.6.5, Rome, Italy) were used to simulate the exercise protocol of a soccer-specific by alternating between periods of standing, walking, jogging, running, and sprinting based on the protocol of Drust et al. (2000) [21]. The percentage of time spent at each speed was based on the time–motion analysis of professional soccer players. Spend about 7% of the match in a standing position, 56% of the match walking (6 km/h), 30% of the match jogging (10 km/h), 4% of the match running (17 km/h), and 3% of the game sprinting (21 km/h) [22] (Figure 3).

### 2.5. Details of Experimental Carbohydrate Conditions

CHO–electrolyte intake experiments are divided into three timings. First, pre-exercise (15 min before beginning the soccer-specific exercise test); second, half-time (immediately upon finishing the first half); and third, mixed (15 min before starting the soccer-specific exercise test and the second half) [23]. The total amount of ingested CHOs was 60 g [7], and in the mixed intake experiment, the amount was divided into half and consumed at intake timing 1 and intake timing 2. The CHO–electrolyte consumed is a product approved for sale (IAMPOTIN Energy gel 80, Seoul, Korea). The components of the product are 20 g of CHOs, less than 5 mg of sodium, and less than 0.5 g of protein. The placebo was ingested 15 min before the soccer-specific exercise test (500 mL). The placebo added an apple flavoring of 1% of the solution to produce apple scents and tastes such as CHO gel (NIF, Food synthetic flavoring, propylene glycol 88.51%, synthetic flavoring). The concentration of CHO–electrolytes was designed to be 12%, and electrolyte intake was the same in all experimental tests (500 mL).

### 2.6. Statistical Analysis 

Results are reported as the mean ± SD. One-way ANOVA was used to analyze multiple comparisons between the experimental tests, analysis of variance followed by the Tukey post-hoc. All statistical significance was considered *p* < 0.05. All statistical analyses were conducted using IBM Windows SPSS Statistics (Version 25, New York, NY, USA).

## 3. Results

### 3.1. Respiratory Gas and Physiology Variable

#### 3.1.1. Respiratory Exchange Ratio

**Figure 4:**
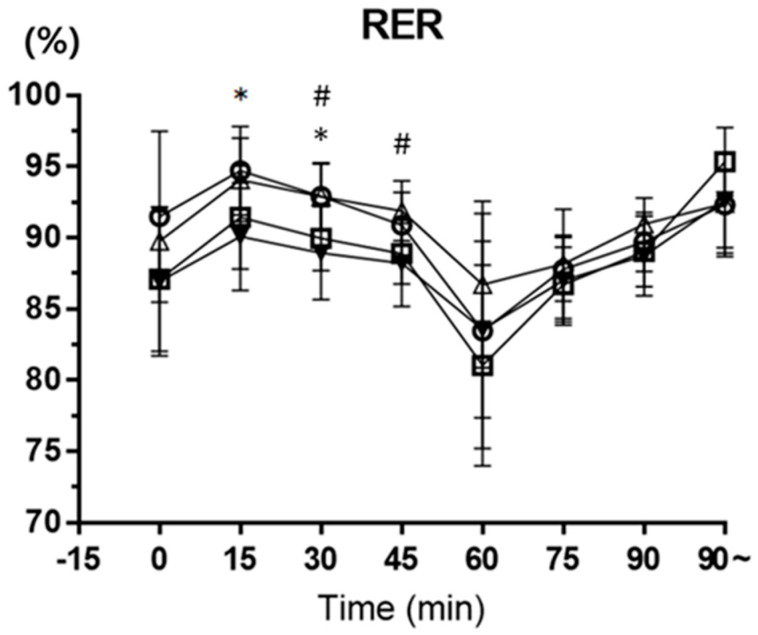
−○− placebo, −□− pre-exercise, −△− half-time, and −▼− mixed. This figure shows the RER collected during soccer-specific intermittent tests. Data are shown in means ± SD, and one−way ANOVA was performed. Tukey test was used to calculate *p* values. Mixed test *, *p* < 0.05 compared with placebo test. Mixed test #, *p* < 0.05 compared with half−time test.

As a result of the one-way ANOVA analysis of variance on RER, the experimental tests showed a significant main effect in means RER 2 (the number after the variable nouns means the number in parentheses in Figure 2, and RER 1 is the means ± SD of RER measured during the warm-up cycle of 15 min., e.g., RER 2: RER cycle 1; RER 5: RER half-time cycle 2; RER 8: RER RTF cycle), RER 3, and RER 4 (*p* < 0.05) (Figure 2). The Tukey post hoc test revealed that RER 2 was significantly lower in the mixed test when compared with the placebo test (*p* < 0.05). Significantly less RER 3 was observed in the mixed test compared with the placebo and half-time test (*p* < 0.05). And the mixed test showed significantly less RER 4 than the half-time test (*p* < 0.05) (Figure 4). The RER (2, 3, and 4) means ± SD are placebo (2: 94.713 ± 3.10, 3: 92.925 ± 2.37), half-time (3: 92.90 ± 2.30, 4: 91.875 ± 2.10), and mixed (2: 90.10 ± 3.79, 3: 88.95 ± 3.29, 4: 88.20 ± 3.03). 

#### 3.1.2. Fat Oxidation

**Figure 5:**
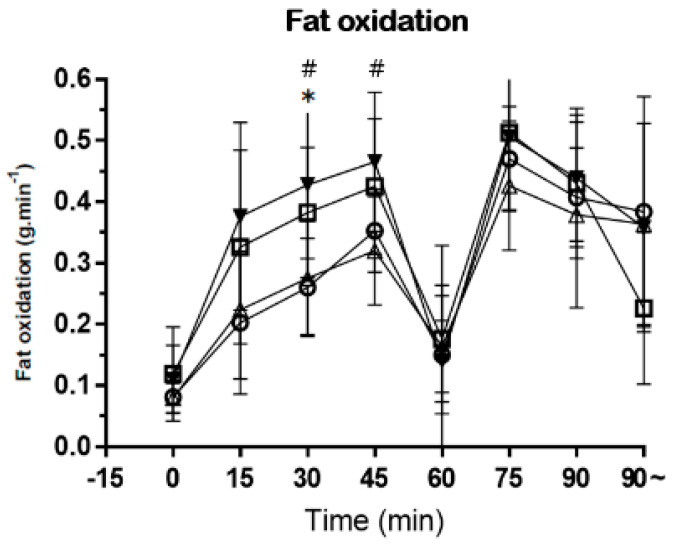
−○− placebo, −□− pre-exercise, −△− half-time, and −▼− mixed. This figure shows the fat oxidation collected during soccer-specific intermittent tests. Data are shown in means ± SD and one−way ANOVA was performed. Tukey test was used to calculate *p* values. Mixed test *, *p* < 0.05 compared with placebo test. Mixed test #, *p* < 0.05 compared with half−time test.

As a result of the one-way ANOVA analysis of variance on fat oxidation, the experimental tests showed a significant main effect in the mean fat oxidation 3 and fat oxidation 4 (*p* < 0.05). The Tukey post hoc test revealed that fat oxidation 3 was significantly higher in the mixed test when compared with the placebo and half-time test (*p* < 0.05). Significantly more fat oxidation 4 was observed in the mixed test than the half-time test (*p* < 0.05) (Figure 5). The fat oxidation (3, 4) means ± SD are placebo (3: 0.26 ± 0.08), half-time (3: 0.28 ± 0.09, 4: 0.32 ± 0.09), and mixed (3: 0.42 ± 0.12, 4: 0.47 ± 0.11). 

#### 3.1.3. Carbohydrate Oxidation

As a result of the one-way ANOVA analysis of variance on CHO oxidation, the experimental tests showed a significant main effect in means pf CHO oxidation 3 (*p* < 0.05). However, the Tukey post hoc test showed no significant differences in CHO oxidation among the tests (*p* > 0.05). The CHO oxidation means ± SD of cycles one to five are placebo test (2.08 ± 0.26), pre-exercise (1.84 ± 0.163), half-time (2.09 ± 0.194), and mixed (1.83 ± 0.102).

#### 3.1.4. Heart Rate

As a result of the one-way ANOVA analysis of variance on HR, the experimental tests showed no significant main effect in means HR (*p* > 0.05). The HR means ± SD of cycles one to five are placebo test (153.46 ± 4.467), pre-exercise (147.63 ± 3.984), half-time (149.83 ± 4.455), and mixed (147.56 ± 4.832).

### 3.2. Exercise Performance

#### 3.2.1. Running Time (RTF)

**Figure 6:**
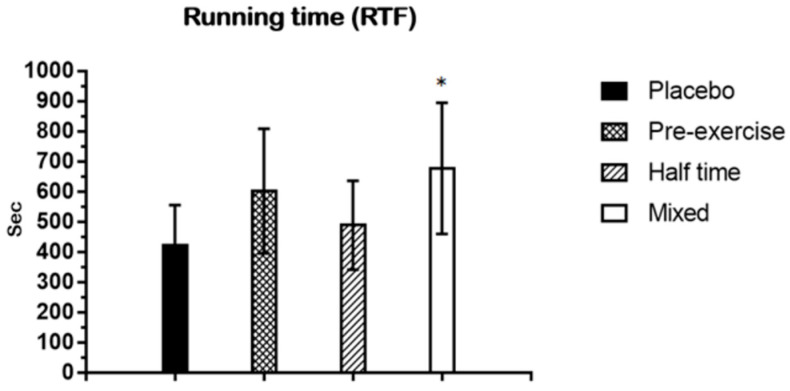
This figure shows the running time collected during RTF. Data are shown in means ± SD and one-way ANOVA was performed. Tukey test was used to calculate *p* values. Mixed test *, *p* < 0.05 compared with Placebo test. Sec: second.

As a result of the one-way ANOVA analysis of variance on running time, the experimental tests showed a significant main effect on the mean running time (*p* < 0.05). The Tukey post hoc test revealed that running time was significantly longer in the mixed test when compared with the placebo test (*p* < 0.05) (Figure 6). The running time means ± SD are placebo (422.13 ± 133.44) and mixed (677.38 ± 217.75).

#### 3.2.2. Distance (RTF)

**Figure 7:**
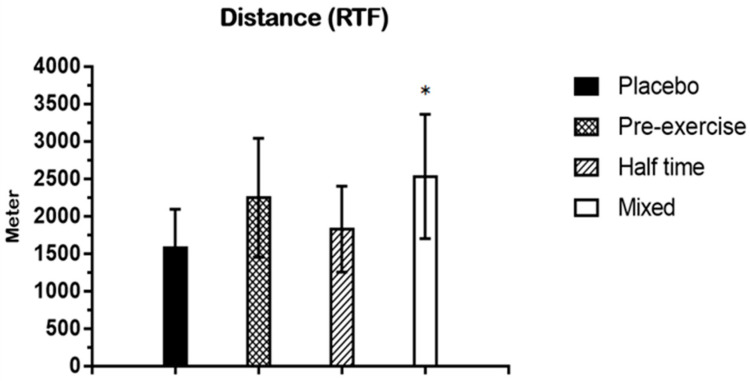
This figure shows the distance collected during RTF. Data are shown in means ± SD and one-way ANOVA was performed. Tukey test was used to calculate *p* values. Mixed test *, *p* < 0.05 compared with placebo test.

As a result of the one-way ANOVA analysis of variance on distance, the experimental tests showed a significant main effect in the mean distance (*p* < 0.05). The Tukey post hoc test revealed that distance was significantly more in the mixed test when compared with the placebo test (*p* < 0.05) (Figure 7). The distance means ± SD are placebo (1577.25 ± 517.02) and mixed (2530.00 ± 832.71). 

### 3.3. Rating of Perceived Exertion

**Figure 8:**
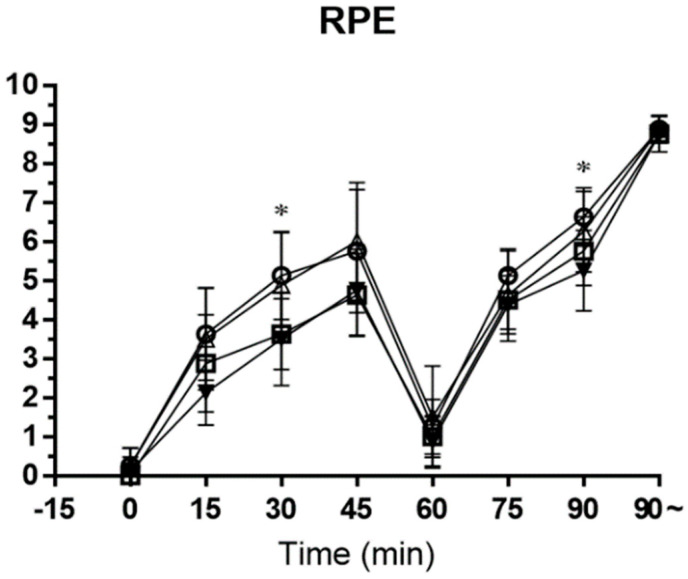
−○− placebo, −□− pre-exercise, −△− half-time, and −▼− mixed. This figure shows the RPE collected during soccer-specific intermittent tests. Data are shown in means ± SD and one-way ANOVA was performed. Tukey test was used to calculate *p* value. Mixed test *, *p* < 0.05 compared with Placebo test.

As a result of the one-way ANOVA analysis of variance on RPE, the experimental tests showed a significant main effect in mean RPE 3 and RPE 7 (*p* < 0.05). The Tukey post hoc test revealed that RPE 3 was significantly lower in the mixed test when compared with the placebo test (*p* < 0.05). Significantly less RPE 7 was observed in the mixed test than the placebo test (*p* < 0.05) (Figure 8). The RPE means ± SD are placebo (3: 5.13 ± 1.13, 7: 5.13 ± 0.74) and mixed (3: 3.50 ± 1.20, 7: 5.25 ± 1.04).

## 4. Discussion

An average human has an RER at rest of around 0.8, although this can vary a bit depending on diet and other factors. During a stress test, RER will typically gradually increase to a peak of about 1.2 (again, variable depending on the individual). As a rule of thumb, a resting RER of about 0.7 indicates that fats are being used as the body’s main fuel source, while 1 means mostly CHOs are being used [24]. The experimental tests showed a significant main effect in the means of RER 2, RER 3, and RER 4 (*p* < 0.05). RER 2 was significantly lower in the mixed test when compared with the placebo test (*p* = 0.05). Significantly less RER 3 was observed in the mixed test compared with the placebo and half-time test (*p* < 0.05). And the mixed test showed significantly less RER 4 than the half-time test (*p* < 0.05). This result is similar to the results of other studies, and it seems that the intake of CHO–electrolyte supports increasing the persistence of exercise by decreasing RER during exercise. 

Fat oxidation increases up to 70% VO_2max_ when stationary but decreases with exercise intensity of about 75% VO_2max_ or higher [25,26]. In this study, fat oxidation 3 was significantly higher in the mixed test when compared with the placebo and half-time test (*p* < 0.05). Significantly more fat oxidation 4 was observed in the mixed test than in the half-time test (*p* < 0.05). Therefore, subjects recognized the mixed and pre-exercise test as having a relatively low exercise intensity compared to the placebo or half-time test in the first half of the exercise protocol. It is commonly believed that increasing fat utilization and sparing CHO are associated with increased endurance [27,28,29]. Previous studies of long-distance athletes have shown that carbohydrates consumed during exercise increase fat oxidation rates during exercise [30]. However, some of the previous studies had different results from this study. Differences in metabolic regulation between high-intensity intermittent and moderate-intensity endurance exercise may be another reason why the substrate oxidation results differ from previous studies. Therefore, a direct comparison between high-intensity intermittent exercise and endurance exercise is laborious. Therefore, this study is meaningful in that it studied fat oxidation following CHO intake by soccer players during prolonged soccer-specific intermittent exercise.

This study showed no significant differences in CHO oxidation among tests (*p* > 0.05). Previous studies researching the effects of CHO intake on exercise performance have focused on prolonged endurance exercise. The results of these studies are mixed, and the same is true of the results of this study [31,32,33]. Muscle glycogen stores decrease as exercise progresses, and blood glucose becomes a progressively more important fuel source [34]. Contrastively, muscle glycogen, not blood glucose or free fatty acids, is the principal fuel source during high-intensity exercise [35]. Therefore, the potential for CHO intake to affect blood glucose and free fatty acid concentration may not be as important during high-intensity intermittent exercise when compared to moderate-intensity exercise [36]. Therefore, this study is meaningful in that it studied CHO oxidation following CHO intake by soccer players during prolonged soccer-specific intermittent exercise. However, further research is needed later to prove the difference between CHO oxidation in intermittent, high-intensity, and persistent exercise.

The exercise protocol of this study was chosen because lower muscle glycogen stores have been associated with decreased distance covered and decreased time spent sprinting during the last 15 min of competitive soccer matches [35]. The last 15 min of soccer matches also appear to be critical to the outcome, as a disproportionate number of goals are scored in this period [37]. Therefore, if a CHO–electrolyte intake resulted in increased reliance on fat and decreased reliance on CHOs during soccer-specific intermittent exercise, muscle glycogen stores during the late stages of the match could be preserved, and the RTF running time may be greater. In this study, running time and distance measured during RTF were significantly higher in the mixed test when compared with the placebo test (*p* < 0.05). In this study, performance improvements were apparent with a CHO supplement at an exercise intensity of 80% V_max_. In particular, the effect was evident in the mixed test in this study. Indeed, this is in agreement with other studies [38,39].

If there is an increased dependence on fat and a decreased reliance on CHO during soccer-specific intermittent exercise, the distance covered on the RTF may be greater. If participants could cover more distance at the same exercise intensity during this period of experimental tests, it is reasonable to assume that CHO–electrolyte intake could affect the outcome [37,40]. These results suggest a potential advantage of endurance performance during soccer-specific intermittent exercise following a mixed intake of CHO–electrolyte. Few studies have evaluated endurance at the end of the soccer-specific intermittent exercise test. Accordingly, it is difficult to make direct comparisons between the performance results from this study and those of previous investigations. However, the previous study provides a basis for comparison. In their study, participants cycled at ~70% VO_2max_ for 120 min and then rode to exhaustion at 100% VO_2max_ [41]. DeMarco et al. (1999) found that the cycling distance to exhaustion was increased in the CHO intake test [41]. However, due to the differences in exercise type, duration, intensity, and character of the performance test, further research is needed to confirm whether CHO–electrolyte provides performance advantages for soccer-specific intermittent exercise performed at the end of a prolonged exercise test.

HR in all experimental tests increased linearly over the course of exercise. This replicates findings from our previous study [42] and from related adult work [43,44]. Although there were no significant differences, the pre-exercise and mixed tests had 5 bpm less HR means ± SD of cycles one to five than the placebo test. In the same intensity of exercise protocol, pre-exercise and mixed tests seem to recognize relatively low intensity compared to placebo tests. The HR tends to be the same as other measurement variables representing exercise intensity, such as RER, fatty oxidation, CHO oxidation, and RPE. The mixed test, in which significantly more RTF running time and distance were collected than the placebo test (*p* < 0.05), showed similar HR. Therefore, CHO–electrolyte intake appears to increase the persistence of exercise intensity. These results are similar to those of previous studies [44,45].

This study found that ratings of perceived exertion using the Borg RPE scale [17] were lower in the mixed test compared to the placebo test (*p* < 0.05). It appears that CHO ergogenic aids result in participants recording a lower RPE score at a given exercise intensity or achieving a higher intensity for a given RPE. However, RPE is not designed to assess central fatigue directly, and therefore, this interpretation should be viewed with caution. Although associative, Using RPE to measure subjective recognition of exertion provides some insight into these fatigue processes [46]. However, RPE is not designed to assess central fatigue directly, and therefore, this interpretation should be viewed with caution. In this study, mixed tests were significantly less in RPE 3 and RPE 7 than placebo tests (*p* < 0.05). Therefore, CHO–electrolyte consumed before exercise can be seen as improving exercise persistence by decreasing the RPE during exercise.

Previous studies in this area have examined continuous, moderate-intensity exercise [39,47]. However, the results of these studies may not be relevant to team sports athletes who are required to perform intermittent activity for extended periods of time. Therefore, this study was unique in that it examined the effects of CHO–electrolyte intake timing on metabolism and performance during prolonged, soccer-specific intermittent exercise. Many studies have investigated the mechanical effects of CHO supplementation on metabolism and performance [48,49,50]. There are many studies that show that carbohydrate intake increases exercise performance, but few have researched the effect of intake timing on exercise performance. In particular, no study has analyzed soccer players by classifying them into three carbohydrate intake timings like this study. Therefore, the results of this study suggest important implications in sports that encompass multiple exercise sessions with limited recovery, such as soccer [51]. In addition, the exercise protocol of this study is a soccer-specific protocol that reflects the exercise characteristics of soccer as much as possible on a treadmill. Therefore, this study is regarded as of high value because it analyzed changes in performance and metabolism according to the timing of carbohydrate intake in soccer players using soccer-specific exercise rules.

However, due to the differences in exercise type, duration, intensity, and character of the exercise test, additional research is needed to prove whether CHO–electrolyte intake provides performance benefits for soccer-specific intermittent exercise performed in the prolonged exercise test. This study used soccer-specific exercise on the treadmill to imitate the demands of a soccer match. Moreover, previous studies have analyzed blood data, but this study has not performed blood collection and analysis [15,52]. Therefore, further studies investigating the effects of CHO intake on metabolism and performance in soccer players will need to analyze blood variables.

## 5. Conclusions

According to this study, in the soccer-specific intermittent exercise test, CHO–electrolyte intake tests showed lower RER, RPE, and increased fat oxidation, running time, and distance than the placebo test. In particular, there were significant differences between the test that mixed consumed CHO–electrolyte and the placebo. In addition, RER and Fat oxidation, which indirectly indicate endurance performance, were higher in the Mixed experiment than in the half-time experiment. Therefore, it can be seen that the intake of CHO–electrolytes improves the performance of soccer players, and the effect varies depending on the timing of CHO–electrolyte intake. In further study, it should be tried to perform more diverse exercise protocols to establish the effects of CHO intake in soccer-specific intermittent exercise. In addition, further studies investigating the effects of CHO intake on metabolism and performance in soccer players will need to analyze blood variables.

## Data Availability

Data available in a publicly accessible repository that does not issue DOIs for publicly available datasets were analyzed in this study. This data can be found here: (https://figshare.com/articles/dataset/Article_data_Effects_of_the_Timing_of_Carbohydrate_Intake_on_Metabolism_and_Performance_in_Soccer_Players_/23919450, accessed on 7 August 2023).
